# The effects of storing and transporting cryopreserved semen samples on dry ice

**DOI:** 10.5935/1518-0557.20160042

**Published:** 2016

**Authors:** David Til, Vera L L Amaral, Rafael A Salvador, Alfred Senn, Thais S de Paula

**Affiliations:** 1 Vale do Itajaí University - UNIVALI - Itajaí/SC, Brazil; 2 F.A.B.E.R Foundation, Lausanne, Switzerland - Lausanne, Switzerland; 3 Sapientiae Institute - São Paulo/SP, Brazil

**Keywords:** Cryopreservation, sperm, dry ice

## Abstract

**Objective:**

This study aimed to test the effects on sperm viability of transporting cryopreserved semen samples on dry ice.

**Methods:**

Twenty normozoospermic semen samples were cryopreserved and divided into five groups. The samples in Group 1 were immersed in liquid nitrogen throughout the experiment in cryogenic storage tanks; the cryopreserved straws in Group 2 were placed in a Styrofoam box containing dry ice and kept under these conditions for 48 hours; the samples in Group 3 were kept for 48 hours on dry ice under the same conditions as the Group 2 samples, and were then moved to a storage tank filled with liquid nitrogen; Group 4 samples were also kept for 48 hours in dry ice storage, and the Styrofoam box containing the samples was shipped by plane to assess the effects of shipping; the samples in Group 5 were shipped together with the Group 4 samples and were placed in a storage tank with liquid nitrogen after spending 48 hours stored on dry ice. After thawing, sperm parameters were analyzed for viability, vitality, and motility; spermatozoa were also tested for mitochondrial activity.

**Results:**

Significant decreases in motility recovery rates (*P*=0.01) and vitality (*P*=0.001) were observed in all groups when compared to the control group. Mitochondrial activity was significantly decreased only in Group 5 (*P*=0.04), as evidenced by greater numbers of sperm cells not stained by reagent 3,3'-diaminobenzidine.

**Conclusions:**

Transportation did not affect the quality of cryopreserved semen samples, but dry ice as a means to preserve the samples during transportation had detrimental effects upon the sperm parameters assessed in this study.

## INTRODUCTION

The advances in reproductive medicine seen over the past decades and the cryopreservation of human semen in particular have significantly aided the clinical management of infertility and the formation of sperm banks. Cryopreservation is an established, proven method used routinely in assisted reproduction laboratories for years (Meseguer *et al*., 2006). It allows semen to be stored at very low temperatures (-196ºC in liquid nitrogen) while preserving sperm function indefinitely (Holt, 2000).

Frozen semen applications currently include preventive sperm storage for reasons such as surgery, radiation therapy, chemotherapy, vasectomy, and exposure to cytotoxic drugs, immunosuppressants, and risk factors. Cryopreservation of human semen for use in assisted reproduction has become a globally recognized procedure (Cavalcante *et al*., 2006).

Over the years, freezing semen at slow, controlled rates has become the method of choice for cryopreservation. Such approach has allowed the use of reproducible cooling rates and yielded effective cell dehydration while precluding the formation of intracellular ice crystals (Gilmore *et al.*, 2000). Despite its effectiveness and satisfactory results, this process induces significant cell stress and imposes extremely unfavorable conditions on sperm in order to maintain its viability (Purdy, 2006). Exposure to these conditions can cause structural damage and functional changes to sperm, such as decreased vitality, motility, speed, and reduced fertilization potential, partly due to plasma membrane integrity disorders and other still unclear factors (Schüffner *et al*., 2008).

The main variables assessed in semen preservation techniques are motility and vitality. Motility indicates the functional competence of sperm cells and vitality is a way to identify living and dead sperm (Cavalcante *et al.*, 2006); the former is one of the most severely affected variables (Watson, 1995). Verza Jr *et al*. (2009) studied the resistance of human sperm to cryoinjury after repeated cycles of freezing and thawing, using the quick method with liquid nitrogen vapor; the authors described a sharp drop in sperm motility after each thawing cycle. However, they failed to take cell membrane damage and acrosome reaction inactivation into account in the process (Watson *et al*., 1992; Devireddy *et al*., 2000).

According to Watson (1995), it is generally accepted that cryopreservation may induce the formation of reactive oxygen species, either through decreases in the effectiveness of the antioxidant protection system (Bilodeau *et al*., 2000) or the release of reactive oxygen species by defective sperm cells or sperm cells killed during the cryopreservation procedure (Bailey *et al*., 2000).

Mitochondrial membrane integrity is an important factor in post-freezing analysis. In the last two decades many authors have looked into mitochondria and the important role they play in sperm physiology and in the production of energy mostly through adenosine triphosphate (ATP). In addition to allowing cell movement (Camera & Guerra, 2008), this organelle present inside mitochondrial DNA (mtDNA) also transcribes several proteins for oxidative phosphorylation. Thus, changes in mitochondrial membrane potential or mutations in mtDNA may interfere with sperm characteristics and male fertility (Câmara & Guerra, 2008).

Various fluorescent markers such as Rhodamine 123, DiOC6 (Wang *et al*., 2003) and JC-1 (Kasimanickam *et al*., 2007) have been used to assess the sperm mitochondrial membrane, suggesting it may be an indicator of sperm functional integrity. Donnelly *et al.* (2000) described a negative correlation between motility and the percentage of sperm cells with mitochondrial dysfunction, both in raw human semen and in samples previously submitted to selection by the Percoll gradient. In contrast, Troiano *et al.* (1998) described a positive correlation between mitochondrial membrane potential and sperm motility.

Specific mitochondrial function assessment techniques, such as mitochondrial membrane integrity testing (O'connell *et al*., 2002; Wang *et al*., 2003; Kasimanickam *et al*., 2007; Hrudka, 1987), became indispensable tools in the evaluation of sperm fertilizing capacity and good indicators of post-freezing sperm quality.

The growing use of donor semen means that more samples are being shipped over long geographical distances. In sperm donation programs and assisted reproduction technology procedures, safe sample transportation across the country is of the utmost importance. Semen specimens are most often transported either on dry ice, at a temperature of about -80ºC, or in cryogenic storage tanks containing liquid nitrogen (-196ºC). Other factors such as temperature fluctuations during sample transportation and handling may negatively affect the quality of thawed sperm. (Carrell *et al*., 1996).

Considering the low number of publications on the adverse effects temperature variations during cryopreserved sample storage and transportation may have on sperm quality after thawing, this study was designed to test the efficiency of storing and transporting cryopreserved semen samples on dry ice for a maximum period of 48 hours and assess semen parameters after thawing.

## MATERIALS AND METHODS

Twenty semen samples sent for laboratory analysis were included in the study. Clarification on the study was provided at the time the patients came to schedule their semen collection appointments, and the individuals who agreed to join the study signed an informed consent form. Only the samples classified as normozoospermic according to the WHO criteria were included in the study (2010).

The samples were conventionally cryopreserved in a 1:1 mixture of semen and Test Yolk Buffer^®^ (TYB, Irvine Scientific, USA). After homogenization, each sample was aliquoted in ten 0.5 ml straws and identified. The straws were taken to a refrigerator at 6ºC (± 2) for 30 minutes, then exposed for 10 minutes to liquid nitrogen vapor (-140ºC) and immediately immersed in liquid nitrogen at -196ºC (RedLara, 2006). After cryopreservation, the samples were divided into five groups, each containing two straws (duplicate).

The samples in Group 1 (CONTROL) were kept immersed in liquid nitrogen (-196ºC) in cryogenic storage tanks throughout the experiment. Group 2 (DRY ICE) samples were first cryopreserved and then transferred to a 10-liter Styrofoam box measuring 45x30x20cm (LxHxW) containing 6 kg of dry ice (-80ºC), an amount deemed sufficient to maintain refrigeration conditions for the duration of the study. The samples remained under these conditions for 48 hours to simulate the maximum travel time needed to cross Brazil by plane. After freezing, the samples in Group 3 (DRY ICE + NITROGEN) were kept for 48 hours on dry ice, under the same conditions as the samples in Group 2, and were then returned to liquid nitrogen, simulating what occurs routinely in clinical settings. Group 4 (TRANSPORTED) samples were kept for 48 hours in dry ice, exactly the same way and under the same conditions as the samples in Group 2, but the Styrofoam box containing the samples was shipped by plane so that the effects related to transportation such as temperature changes and adverse effects could be analyzed. The samples in Group 5 (TRANSPORTED + NITROGEN) were shipped together with the samples in Group 4 and were stored to liquid nitrogen after 48 hours of storage on dry ice.

The samples in groups 4 and 5 were shipped by plane between the airports of Navegantes, São Paulo and Florianópolis, and were sent back to the originating laboratory for analysis. The samples were sent back to the laboratory in less than 48 hours.

After 48h, all the samples (two vanes per group) were removed from dry ice (-80ºC) and/or liquid nitrogen (-196ºC) and kept for 25 minutes at 37ºC, and then in modified HTF medium (Irvine Scientific - IRVINE^®^) supplemented with 10% synthetic serum (Synthetic Serum Substitute; Irvine Scientific - IRVINE^®^) in a 1:1 mixture centrifuged for 8 minutes at 1500rpm (800G) and resuspended in 250µL of the same medium. After thawing, semen samples were assessed for motility and vitality (stained with eosin) according to the REDLARA Manual (2006), and mitochondrial activity as determined by the method described by Hrudka (1987).

## RESULTS

**Figure 1:**
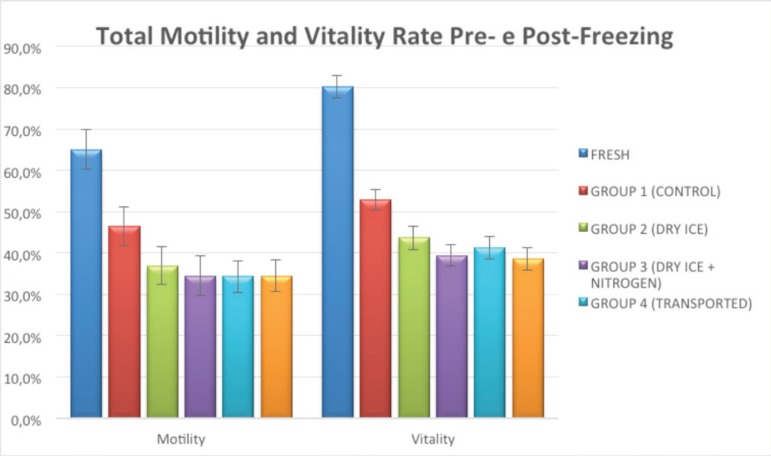
Mean percent distribution of motility rates and vitality for samples from different groups before and after freezing.

Compared to fresh samples, all groups had reduced vitality and motility, as shown in Table 1 and Figure 1.

**Table 1:** Mean percent value (± standard error) of total motility and vitality recovery rates of samples from different groups before and after freezing.

	Before Freezing (COOL)	After Freezing				
		**GROUP 1** **(CONTROL)**	**GROUP 2** **(DRY ICE)**	**GROUP 3** **(DRY ICE +NITROGEN)**	**GROUP 4(TRANSPORTED)**	**GROUP 5(TRANSPORTED** **+ NITROGEN)**
MOTILITY (%)	65.1 ± 2.3	46.6 ± 3.2	36.9 ± 3.1	34.5 ±3.1	34.4 ± 3.2	34.6 ± 2.5
VITALITY (%)	80.3 ± 2.2	53.0 ± 2.8	43.8 ± 2.7	39.5 ± 2.7	41.4 ± 2.7	38.6 ± 2.8

Post-freeze recovery rates (thawed/fresh samples) were lower in Groups 2-5 than in Group 1 (Control).

Different levels of mitochondrial activity were observed when the samples in all groups were compared to the ones in group 4 (*P*=0.04), as shown in Table 3.

**Table 2:** Mean percent value (± standard error) of total motility and vitality recovery rates for samples from different  groups after freezing.

	GROUP 1 (CONTROL)	GROUP 2 (DRY ICE)	GROUP 3 DRY ICE + NITROGEN)	GROUP 4 (TRANSPORTED)	GROUP 5 (TRANSPORTED + NITROGEN)
MOTILITY (%)	71.9 ± 4.7^a^	56.7 ± 4.6^b^	52.7 ± 4.5^b^	52.8 ± 4.8^b^	53.0 ± 3.7^b^
VITALITY(%)	63.9 ± 2.6^a^	53.7 ± 2.4^b^	48.6 ± 2.8^b^	50.6 ± 2.5^b^	46.7 ± 2.7^b^

*Different letters in the same row differ. *P* <0.05.

**Table 3:** Mean percent value (± standard error) of cells in Class I, II, III and IV, for 3,3’-diaminobenzida staining to assess mitochondrial activity in spermatozoa of fresh samples and different groups tested.

	PRE-FREEZ ING (COOL)	Post-Freeze				
		GROUP 1 (CONTROL)	GROUP 2 (DRY ICE)	GROUP 3 (DRY ICE + NITROGEN)	GROUP 4 (TRANSPORTED)	GROUP 5 (TRANSPORTED + NITROGEN)
Class I (%)	25.8 ± 1.6^a^	25.3 ± 1.8^a^	23.7 ± 1.4^a^	24.4 ± 1.5^a^	24.3 ± 1.7^a^	24.5 ± 1.4^a^
Class II (%)	50 ± 1.5^a^	49.2 ± 1.5^a^	49.4 ± 1.2^a^	48.8 ± 1.3^a^	48.2 ± 1.4^a^	48.3 ± 1.3^a^
Class III (%)	15.4 ± 1.1^a^	14.8 ± 1.2^a^	15.9 ± 1.1^a^	15.6 ± 1.0^a^	15.5 ± 1.1^a^	14.8 ± 1.1^a^
Class IV (%)	8.8 ± 0.8^a^	10.6 ± 1.0^a^	10.9 ± 0.8^a^	11.1 ± 0.7^a^	11.9 ± 0.9^a^	12.3 ± 0.7^b^

*Different letters in the same row differ. *P *<0.05.

## DISCUSSION

Cryopreservation induces physical and chemical damage to cell membranes, exposing sperm cells to extremely unfavorable conditions and adversely affecting their viability (Purdy, 2006), as shown by the comparison of pre and post-freeze motility rates and vitality (Carrell *et al.*, 1996).

Decreased levels of motility and vitality were observed in this study after thawing. However, such decrease had varying orders of magnitude between donors, as also found by Steinberger & Smith (1973), showing that the response to cryopreservation of sperm cells from different individuals is significantly different.

**Figure 2:**
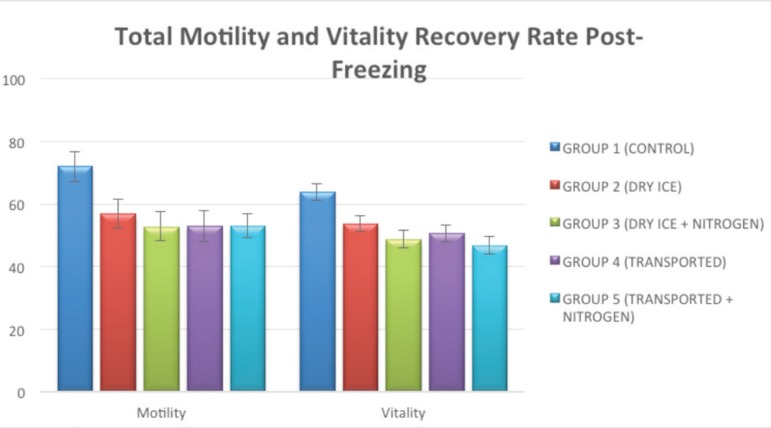
Mean percent value (± standard error) of total motility and vitality recovery rates of samples from different groups before and after freezing.

According to Agarwal (2000), sperm cell post-cryopreservation motility rates and vitality may be decreased by 25% to 75%, mainly due to the stress to which these cells are subjected during the cryopreservation process, derived chiefly from cell dehydration, high solute concentrations, recrystallization (Petrunkina, 2007), and changes in plasma membrane integrity, thus leading to sperm cell functional changes and structural damage (Shuffner *et al.,* 2008).

Carrell *et al.* (1996) correlated variations in the rates of recovery of sperm cell vitality and motility with sample initial quality, handling conditions, and protocol used. In addition to these factors, storage conditions also play an important role, since these samples are often transported and exposed to temperature variations, quick exposure to ambient temperature during tank changes, and longer exposures to higher temperatures when they are shipped over long distances in dry ice (-80ºC).

Brotherton (1990) reported that enzymes related to cell aging become virtually inactive at temperatures below -70ºC; however, during transportation temperatures tend to be slightly higher than that of dry ice, which may allow for the activation of enzymes activation and the removal of cells from a latent state.

Recrystallization is an important process that occurs during heating, generally at temperatures around -87oC. Samples kept on dry ice for prolonged periods of time are at increased risk of forming intracellular ice crystals, which can cause cell and membrane damage and directly affect the viability and motility of sperm cells (Karow, 1974).

Motility (*P* = 0.01) and vitality (*P* = 0.0001) recovery rates were significantly different in the samples in Group 1 (control) compared to the samples in the other groups. However, the samples in Groups 2, 3, 4 and 5 were not different when compared to each other, showing that the key factor for decreased cell vitality and motility was not directly correlated with transportation, but with temperature variation, a variable associated with adverse effects as described by Carrell *et al*. (1996).

The comparison of samples in Groups 2 and 4 - stored in dry ice - against samples in Groups 3 and 5 - stored in liquid nitrogen - failed to yield significant differences, indicating that the damage suffered by sperm cells may be related to the recrystallization process that occurs when the samples are heated, such as when they are removed from liquid nitrogen (-196ºC) and exposed to dry ice (-80ºC), which does not occur in the reverse process when they are stored back in liquid nitrogen (Table 1 and 2).

Sherman (1963) and Trummer *et al.* (1998) found similar results in their studies. Significant decreases in motility were seen in the samples kept in dry ice, in comparison to samples stored in liquid nitrogen. According to Trummer *et al*. (1998), this finding may be related to the physical and chemical characteristics of the cryoprotectant solution, which in most cases reaches a solid state at temperatures around -75ºC, in a phase where crystals may form. On the other hand, at lower temperatures there is more stability and less risk of crystals forming and subsequent cell damage.

In terms of mitochondrial activity, no significant differences were observed between groups for cells in classes I, II and III, even when compared to fresh samples. This shows that, despite the decrease in motility and vitality, cellular respiration was still active in sperm cells. Valle (2007) reported similar findings, indicating that even though cells with damaged plasma membranes stained with eosin, vitality testing proved that they still had an active metabolism and should therefore not be considered dead.

Eosin staining was used to assess sperm cell vitality in this study. Greater numbers of stained cells were seen in Groups 2, 3, 4 and 5 than in Group 1 (control), possibly because of changes in membrane permeability which allowed the dye to penetrate the cells as a result of stress to which the samples were submitted due to temperature variations, since no changes in mitochondrial activity were observed (Benson *et al*., 2012).

The positive correlation between sperm motility and cell respiration and energy metabolism described by Amann (1989) was not observed in our study, indicating that the sharp decline in motility was related to factors other than cellular respiration. Valle (2007) suggested that decreased motility might be related to plasma membrane integrity, which might explain the results found in this study.

No changes were seen between Groups 1, 2, 3, 4, 5 and fresh samples, for cells categorized as Class I, II and III. Cells in class IV, i.e., cells with no mitochondrial activity, were statistically different (*P*=0.04) from samples in Group 5 and the other groups. This indicates that this group was exposed to conditions that were more harmful to sperm cells than the other groups, thus negatively affecting their mitochondrial activity.

## CONCLUSION

The results presented in this study showed that transportation does not affect semen parameters. Storage of semen samples on dry ice, however, may potentially affect sperm quality. Further studies are required to analyze other means to transport sperm cells.

## References

[r1] Agarwal A (2000). Semen banking in patients with cancer: 20 years
experience. Int J Androl.

[r2] Amann RP (1989). Can the fertility potential of a seminal sample be predicted
accurately?. J Androl.

[r3] Benson JD, Woods EJ, Walters EM, Cristere JK (2012). The cryobiology of spermatozoa. Theriogenology.

[r4] Bilodeau JF, Chatterjee S, Sirard MA, Gagnon C (2000). Levels of antioxidant defenses are decreased in bovine
spermatozoa after a cycle of freezing and thawing. Mol Reprod Dev.

[r5] Brotherton J (1990). Cryopreservation of human sperm. Arch Androl.

[r6] Câmara DR, Guerra MMP (2008). Mitocôndria espermática: além da
síntese de adenosina trifosfato (ATP). Rev Bras Reprod Anim.

[r7] Carrell DT, Wilcox AL, Urry RL (1996). Effect of fluctuations in temperature encountered during handling
and shipment of human cryopreserved semen. Andrologia.

[r8] Cavalcante MB, Duarte ABG, Araújo DO, Teles A (2006). Criopreservação de sêmen humano:
comparação entre métodos de congelação e
tipos de envase. Rev Bras Ginecol Obstet.

[r9] Devireddy RV, Swanlund DJ, Roberts KP, Pryor JL (2000). The effect of extracelular ice and cryoprotective agents on the
water permeability parameters of human sperm plasma membrane during
freezing. Hum Reprod.

[r10] Donnelly ET, O'connell M, Mcclure N, Lewis SE (2000). Differences in nuclear DNA fragmentation and mitochondrial
integrity of semen and prepared human spermatozoa. Hum Reprod.

[r11] Gilmore JA, Liu J, Woods EJ, Peter AT, Critser JK (2000). Crioprotective agent and temperature effects on human sperm
membrana permeabilities: convergence of theorical and empirical approaches
for optimal cryopreservation methods. Hum Reprod.

[r12] Holt WV (2000). Fundamental aspects of sperm cryobiology: the importance of
species and individual differences. Theriogenology.

[r13] Hrudka F (1987). Citochemical and ultracytochemical demonstratio of
cytochrome-coxidase in spertmatozoa and dynamics of its changes accompanying
aging or induced by stress. Int J Androl.

[r14] Karow AM, Karow AM, Abouna GJM, Humphries AL (1974). Cryopreservation: Biophysical and chemical
considerations. Organ Preservation for Transplantation.

[r15] Kasimanickam R, Kasimanickam V, Pelzer KD, Dascanio JJ (2007). Effect of breed and sperm concentration on the changes in
structural, functional and motility parameters of ram- lamb spermatozoa
during storage at 4º C. Anim Reprod Sci.

[r16] Meseguer M, Molina N, Garcia-Velasco J, Remohí J, Pellicer A, Garrido N (2006). Sperm Cryopreservation inoncological patients: a 14-year
follow-up study. Fertil Steril.

[r17] O'connell M, Mcclure N, Lewis SE (2002). The effects of cryopreservation on sperm morphology, motility and
mitochondrial function. Hum Reprod.

[r18] Petrunkina A (2007). Fundamental aspects of gamete cryobiology. J Reprod Med Endocrinol.

[r19] Purdy PH (2006). A review on goat sperm cryopreservation. Res Small Rumin.

[r20] RED LARA- Red Latinoamericana De Reproducción
Asistida (2006). Manual de Procedimentos: Laboratório de Reprodução
Assistida.

[r21] chüffner A, Morshedi M, Oehninger S, Carvalho NS, Oliveira MTCR, Placido T, Urbanetz AA (2008). Apoptosis and lipid peroxidation before and after
cryopreservation. Reprod Clim.

[r22] Sherman JK (1963). Improved methods of preservation of human spermatozoa by freezing
and freeze-drying. Fertil Steril.

[r23] Steinberger E, Smith KD (1973). Artificial insemination with fresh or frozen semen. A comparative
study. JAMA.

[r24] Troiano L, Granata AR, Cossarizza A, Kalashnikova G, Bianchi R, Pini G, Tropea F, Carani C, Franceschi C (1998). Mitochondrial membrane potential and DNA stainability in human
sperm cells: a flow cytometry analysis with implications for male
infertility. Exp Cell Res.

[r25] Trummer H, Tucker K, Young C, Kaula N, Meacham RB (1998). Effect of storage temperature on sperm
cryopreservation. Fertil Steril.

[r26] Valle RR (2007). Collection, analysis and cryopreservation of semen from a model species,
the common marmoset (Callithrix jacchus).

[r27] Verza JS, Feijo CM, Esteves SC (2009). Resistance of human spermatozoa to cryoinjury in repeated cycles
of thaw-refreezing. Int Braz J Urol.

[r28] Wang X, Sharma RK, Gupta A, George V, Thomas AJ, Falcone T, Agarwal A (2003). Alterations in mitochondria membrane potential and oxidative
stress in infertile men: a prospective observational study. Fertil Steril.

[r29] Watson PF (1995). Recent developments and concepts in the cryopreservation of
spermatozoa and the assessment of their post-thawing
function. Reprod Fertil Dev.

[r30] Watson PF, Critser JK, Mazur P, Templeton AA, Drife JO (1992). Sperm preservation: fundamental cryobiology and practical
implications. Infertility.

[r31] WHO- World Health Organization (2010). Laboratory manual for the examination of human semen and semen-cervical
mucus interaction.

